# The incidence and features of Delphian lymph node involvement in patients with papillary thyroid carcinoma

**DOI:** 10.1186/s12893-022-01742-5

**Published:** 2022-08-20

**Authors:** Abbas Alibakhshi, Saman Sheikhi, Seyed Mostafa Meshkati Yazd, Ali Ardekani, Keivan Ranjbar, Reza Shahriarirad

**Affiliations:** 1 Department of Surgery, Imam Khomeini Hospital Complex, Faculty of Medicine, Tehran University of Medical Sciences, Tehran, Iran; 2 Department of Surgery, Faculty of Medicine, Shahid Madani Hospital, Alborz University of Medical Sciences, Alborz, Iran; 3 School of Medicine, Shiraz University of Medical Sciences, Shiraz, Iran; 4 Thoracic and Vascular Surgery Research Center, Shiraz University of Medical Science, Shiraz, Iran

**Keywords:** Papillary thyroid carcinoma, Sentinel lymph node, Delphian lymph node, Lymphatic metastasis

## Abstract

**Introduction:**

In papillary thyroid cancer patients, the extent of dissection is still a matter of debate. Evaluating Delphian lymph nodes (DLNs) during the surgery has been speculated as a valuable tool to determine the extent of dissection. Herein, we aimed to evaluate the incidence and features of DLNs involvement in patients with papillary thyroid carcinoma.

**Method:**

We conducted this cross-sectional study among surgical cases of papillary thyroid cancer. Patients were divided based on their DLNs involvement status. Their age, gender, location of the mass, lymphatic involvement, tumor size, tumor characteristics, pathology report, and operation note features were compared between the two groups. Definitive pathology slides of the patients were evaluated regarding DLN features.

**Results:**

Of the 61 patients (mean age: 38.2 ± 12.0), 45 (73.8%) were females. In 13 (21.3%) patients, DLNs involvement was reported. A statistically significant relationship was noted between DLNs involvement and other lymph nodes' involvement on the same side of the mass (P < 0.001), the opposite side (P = 0.041), and also central lymph nodes (P < 0.001). Vascular invasion was also significantly higher among patients with DLNs involvement (P = 0.012).

**Conclusion:**

Since DLNs involvement is significantly associated with extensive nodal involvement, intraoperative evaluation of DLNs is recommended to establish the extent to which dissection should be performed.

## Introduction

Delphian lymph nodes (DLNs) are part of the level VI nodes of the neck. They are located anterior to the cricothyroid membrane and are the first nodes that the surgeon encounter during total thyroidectomy [1]. DLNs involvement has long been assumed to predict a decrease in survival rate or an increase in the chance of relapse for head and neck cancers [2–4]. The pretracheal lymph nodes are positioned in front of the isthmus to be drained out toward the mediastinum, and the DLNs are drained out toward the lateral neck following the superior thyroid artery. However, the exact mechanism of lymph node (LN) metastasis is still under debate [4–6]. The involvement of DLNs in laryngeal and hypopharyngeal cancers has been established as a poor prognostic factor [7–9]. However, thyroid cancers, as the most common type of endocrine malignancies with papillary thyroid cancer (PTC) considered the most prevalent one, had miscellaneous results regarding the involvement of DLNs and their predictive value [10], especially when considering level IV central LN dissection. [4]

The pretracheal lymph nodes are situated in front of the isthmus to be drained out into the mediastinum, and the DLNs are drained out toward the lateral neck following the superior thyroid artery, but the exact mechanism of lymph node metastasis is still under question [15–17]. DLN metastasis is recognized as a predictor of extensive LN metastasis, high recurrence incidence, and an elevated mortality rate, particularly in laryngeal cancer. Although according to the existing guidelines, ultrasonography before surgery is recommended to determine the extent of lymph node involvement in PTC [11], we should acknowledge the limitations of ultrasonography in recognizing central lymph node involvement due to the anterior position of the thyroid gland relative to the central lymph nodes, and shadow of the bones [12, 13]. The results of ultrasonography are widely dependent on the physician's competency. Furthermore, central and lateral lymph node involvement has been reported to be 12–81% and 3.1–65.4% in PTC patients, respectively, which is notably high [14, 15]. Hence, it is significant to determine possible predictors of lymph node metastasis, such as DLNs involvement, to act as a marker for extensive nodal involvement [13].

Regarding PTC, on the one hand, some researchers claimed that DLN involvement could be found without other nodes' involvement; thus, it is not a sign of extensive involvement [16]. On the other hand, some studies suggested a predictive role for DLNs as they can signify extensive nodal and vascular involvement [3, 4, 17]. Of note, DLN's involvement is of paramount significance in determining the extent of dissection, treatment planning, and prognosis of the patients [18]. In the present study, we aimed to evaluate the prevalence and determine the features DLNs involvement in PTC patients.

## Method

We conducted a cross-sectional study between 2018 and 2020 in a tertiary care center in Tehran, the capital of Iran. The study protocol was approved by the ethics committee of the Tehran University of Medical Sciences (IR.TUMS.IKHC.REC.1398.04). To determine the sufficient sample size for this study, according to the previous studies and assuming the prevalence of 20% for DLN involvement in papillary thyroid carcinomas, confidence interval 95%, d = 0.05, and z = 1.96, and given the yearly number of the cases in our center (80 papillary thyroid cancer cases) a sample size of 61 was considered as sufficient.

Patients diagnosed with PTC underwent total thyroidectomy and central LN dissection, including prelaryngeal, pretracheal, and paratracheal dissection, by an endocrinology surgeon [19]. The DLNs, also known as the prelaryngeal or cricothyroid nodes, are located in the fascia above the thyroid isthmus and lie between the cricoid and thyroid cartilages. After releasing the pyramidal lobe, the lymph tissues of this area were resected by preserving the cricothyroid membrane and sent for definitive pathology.

All medical records of patients who had pathological confirmation for PTC during the mentioned period were reviewed. Two independent expert pathologists confirmed the definite permanent pathological diagnosis. All patients received standard care according to the available guidelines. The inclusion criteria were: Definitive pathologically confirmed PTC patients who underwent surgery between 20 and 70 years old, with sufficient medical records and DLN evaluation data. The patients were excluded from the study if they had incomplete records or other diagnoses than PTC.

The data were imported to a predesigned Excel (Microsoft, Redmond, WA, U.S.) sheet comprising the patients' age, gender, location of the mass, lymphatic involvement, tumor size, tumor characteristics, pathology report, and operation note features. We divided the patients based on DLNs involvement status. We used SPSS version 21 software to analyze the data utilizing the T-test, Chi-square, and Fischer exact test. A P-value less than 0.05 was considered significant.

## Results

In this study, a total of 200 cases of PTC were evaluated, in which only 61 patients had available permanent pathology slides along with DLN sections. The mean age of the patients was 38.2 (SD: 12.0; range: 20–69) years, and 73.8% were females. Also, the average size of the tumor was 2.1 (SD: 1.6; range: 0.2–7.9) centimeters. Among the patients, 13 (21.3%) had DLNs involvement, while 48 (78.7%) had no involvement. The majority of PTCs were on the right side (50.8%). The rate of lymphatic involvement on the same side of the mass was 14.8%, while on the opposite side was 6.6%. The most frequent pathology was classical papillary thyroid tumor (78.7%), followed by follicular type (14.8%). Table 1 demonstrates the clinical features of the patients in our study.

**Table 1:** Clinical features of patients with papillary tumor

Variable		Total; N = 61	Delphian lymph node involvement		P-value*
			Positive; *n* = *13*	Negative; *n* = *48*	
Age (years); mean ± SD		38.2 ± 12.0	39.2 ± 13.2	37.9 ± 11.7	0.743
Age group; n (%)	≤ 30	22 (36.1)	5 (38.5)	17 (35.4)	0.803
	31–40	18 (29.5)	3 (23.1)	15 (31.3)	
	41–50	10 (16.4)	3 (23.1)	7 (14.6)	
	51–60	8 (13.1)	1 (7.7)	7 (14.6)	
	> 60	3 (4.9)	1 (7.7)	2 (4.2)	
Gender; n (%)	Male	16 (26.2)	4 (30.8)	12 (25.0)	0.728
	Female	45 (73.8)	13 (69.2)	36 (75.0)	
Location of mass; n (%)	Right	31 (50.8)	8 (61.5)	23 (47.9)	0.240
	Left	21 (34.4)	1 (7.7)	20 (41.7)	
	Bilateral	9 (14.8)	4 (30.8)	5 (10.4)	
Lymphatic involvement; n (%)	Same side of mass	9 (14.8)	8 (61.5)	1 (2.1)	** < 0.001**
	The opposite side of mass	4 (6.6)	3 (23.1)	1 (2.1)	**0.041**
	Central lymph node	15 (26.3)	9 (69.2)	6 (12.5)	** < 0.001**
Tumor size (centimeter); mean ± SD		2.1 ± 1.6 (0.2–7.9)	2.4 ± 1.3	2.0 ± 1.7	0.428
Tumor size group; n (%)	≤ 1 cm	17 (29.8)	3 (27.3)	14 (30.4)	1.000
	> 1 cm	40 (70.2)	8 (72.7)	32 (69.6)	
Tumor characteristics; n (%)	Multifocal	15 (24.6)	6 (46.2)	9 (18.8)	0.067
	Vascular invasion	23 (37.7)	9 (69.2)	14 (29.2)	**0.012**
	Capsule invasion	30 (49.2)	6 (46.2)	24 (50.0)	0.806
	Perineural invasion	5 (8.2)	1 (7.7)	4 (8.3)	1.000
Pathology; n (%)	Classical	48 (78.7)	12 (92.3)	36 (75.0)	0.875
	Follicular	9 (14.8)	1 (7.7)	7 (16.7)	
	Medullary/papillary microcarcinoma	1 (1.6)	0 (0)	1 (2.1)	
	Tall cell	1 (1.6)	0 (0)	1 (2.1)	
	Tall cell and follicular	1 (1.6)	0 (0)	1 (2.1)	

*SD* standard deviation

Bold value indicator of significant association

*Chi-square/Fisher’s exact test or independent sample t-test

The positive predictive value (PPV), negative predictive value (NPV), sensitivity, and specificity for lymphatic involvements in the same side of the mass, opposite side of the mass, central lymph nodes, and vascular invasion based on DLN involvement status is presented in Table 2.

**Table 2:** The potency of Delphian lymph node involvements* in predicting other parts' involvements

	Same side of mass	Opposite side of mass	Central lymph node	Vascular invasion
PPV	8/13; 61.5%	3/13; 23.1%	9/13; 69.2%	9/13; 69.2%
NPV	47/48; 97.9%	47/48; 97.9%	42/48; 87.5%	34/48; 70.8%
Sensitivity	8/9; 88.9%	3/4; 75.0%	9/15; 60.0%	9/23; 39.1%
Specificity	47/52; 90.4%	47/57, 82.5%	42/46; 91.3%	34/38; 89.5%

*PPV* positive predictive value, *NPV* negative predictive value

*The values are calculated for significant findings

Despite the higher frequency of DLN involvement in women, this difference was not statistically significant (P = 0.728). The right masses had the highest frequency of DLN involvement, which was statistically significant (P = 0.024). Also, there was a statistically significant relationship between DLN involvement and other lymph nodes' involvement on the same side of the mass (P < 0.001), the opposite side (P = 0.041), and also central lymph nodes (P < 0.001). Vascular invasion was also significantly higher among patients with DLN involvement (P = 0.012).

The mean age of patients with DLN involvement was 39.2 ± 13.2 years, and the mean mass size was 1.3 ± 2.4 cm. Based on the independent t-test, no statistically significant relationship was observed between age and mass size with DLN involvement (P = 0.428 and 0.743, respectively).

We experienced no complications such as vascular and nerve damage among the patients in our study. No cases of symptomatic or clinical hypocalcemia were observed among our patients. Furthermore, patients with transient hypocalcemia based on laboratory calcium tests had calcium levels above 7.5 mg/dL. These patients were discharged with oral calcium supplements and followed up with laboratory calcium assessment every 48 h, in which medication was adjusted or discontinued accordingly.

## Discussion

In the present study on PTC patients, we found a significant association between DLNs involvement and lymphatic involvement in the same side of the mass, opposite side of the mass, and central lymph nodes. Also, the vascular invasion was observed to be significantly associated with DLNs involvement. The incidence of DLNs metastasis in PTC patients ranges from 8.2 to 24.8%, according to Wang et al. meta-analysis [20]. In our study, 23.1% of the patients had DLN involvement, which is in line with previous investigations.

Previous studies attempted to determine whether DLNs involvement can be a predictive factor for extensive nodal involvement in PTC and subsequently guide the surgeon to determine the extent of dissection. Since DLNs receive lymphatic drain from almost all parts of the thyroid gland [6], it can be assumed that DLN is an appropriate marker for extensive nodal involvement. A meta-analysis by Huang et al. [21] showed that in a positive DLN, the risk of having metastasis to central and lateral lymph nodes is significantly higher than in DLN- negative patients. Although after acknowledging their limitations and heterogeneity among the included studies, they suggested frozen section evaluation of DLNs during the operation, and if the nodes were positive, they suggested central lymph node dissection and comprehensive assessment of lateral lymph nodes. These data align with our study, which showed a significant association between DLN involvement with lymphatic involvement in the same side of the mass, opposite side of the mass, and central lymph nodes.

Also, in contrast with several previous studies [18, 20, 22, 23], we found no significant relationship between DLNs involvement with poor prognostic factors like higher age, male gender, larger tumor size, and mass location. More recently, studies report the association between PTC location and DLNs features. A study by Zaho et al. [24] among 1305 patients with isthmus PTC found that the rate of lymph node positivity was 14.9%. Another study by Liu et al. [25] on 522 patients with unilateral PTC reported a 25.2% metastasis rate in DLNs. Both studies align with our results, demonstrating that central and lateral lymph node metastasis is significantly associated with DLNs positivity; Thus, they suggested a frozen section of the DLNs during the surgery to determine the extent of dissection. However, these results need to be interpreted with caution, and further longitudinal studies are required to confirm these findings.

DLN is among the initial locations encountered during total thyroidectomy and based on its drainage properties, provides a valuable prediction of the required extension of dissection in PTC patients [26]. By utilizing this checkpoint as a predictive measure for our surgery, further exploration that can cause nerve and artery damage can be avoided. However, although DLN involvement may be a specific factor, its involvement may not be detected during surgery due to its rarity, resulting in a misdiagnosis and mismanagement in total thyroidectomy patients.

Researchers have criticized the systematic application of frozen sections in thyroid surgery because of its low sensitivity and high rates of false-negative results [27]. Real-time diagnosis of the DLN status can be easily guided by an intraoperative frozen section evaluation [28]. Studies have also advised performing a central compartment neck dissection if the results of intraoperative frozen section examination indicate that DLN is positive. Levels II, III, and IV's ipsilateral lateral LN should be carefully assessed [28]. However, more accurate and thorough individual examination of each sub-lateral level's LN combining clinicopathologic aspects will still be required [29, 30]. Iyer et al. observed that the occurrence of DLN metastases in thyroid tumours is correlated with central and lateral neck LN metastasis and the requirement of thorough LN removal and frozen section biopsy during surgery [31]. Since the DLN is encountered near the region of the cricoid or thyroid cartilage, in cases where the frozen section of the DLN is positive and when a central neck dissection is not initially planned, positivity should trigger a central neck dissection to be performed [26]. Kim et al. suggested that in addition to restricting contralateral central neck LN dissection to specific cases, performing thyroid lobectomy and ipsilateral central neck LN dissection as a result of frozen section biopsy results during surgery may be sufficient for treating PTC patients [1].

We should acknowledge the shortcomings of the frozen section in determining the extent of invasion. Thus, as we applied in our study, permanent pathology was suggested to be used as an indicator of capsular or extra-capsular invasion; however, a frozen section may disintegrate the tissues that should be sent for permanent pathology [27]. The frozen section is proposed to be helpful for those who have suspicious fine needle aspiration for malignancy before surgery [32, 33]. Given the higher risk of post-operative complications of central lymph node dissection during thyroidectomy, previous studies have suggested pre- or intraoperative molecular methods to determine the extent of surgery or only performing dissection for high-risk groups [27, 34–36]. In our study, to achieve more accurate and reliable results, the lymph tissues of the pre-laryngeal region were resected and sent for definitive pathology, while DLNs are a possible proxy for extensive nodal and vascular invasion. However, as an alternative method during surgery, the lymph tissues of this area can be sent for a frozen section, and based on the involvement or non-involvement of the DLNs, a decision for further lymph dissection, including completing central lymphadenectomy can be made. Thus, future studies should adopt various diagnostic methods intraoperatively to determine DLN involvement. We presume that the advantage of this technique in comparison with others is that due to the anatomical location of DLNs, in dissection and resection of lymphatic tissues in this area, the possibility of damage to arteries and nerves is low and extensive dissection will not be required in all cases.

In terms of vascular invasion, previous studies indicated a significant relationship between DLNs positivity and invasion of vascular or lymphovascular structures [1, 20], which is in line with our results.

This study had several limitations. Females outnumbered males in this study; however, this is justifiable since PTC is more prevalent in females. Second, although we tried to provide a sufficient sample size of our patients, our data is limited due to the small sample size and the fact that this was a single-center study. Third, given the study's cross-sectional design, it was not possible to establish the survival or recurrence rate of the patients.

Future prospective studies should focus on the relationship between specific tumor locations, extensive nodal involvement, survival rate, recurrence, and prognosis of patients with and without DLNs involvement, to portray a more obvious picture of this topic.

## Conclusion

DLNs metastasis in PTC patients could accompany extensive nodal and vascular involvement. Therefore, a comprehensive evaluation of DLNs during the surgery and determining the extent of dissection based on the positivity and negativity of the DLNs is justifiable.

## Data Availability

The datasets used and analyzed during the current study are available from the corresponding author on reasonable request and with permission of the Research Ethics Committee of the School of Medicine-Tehran University of Medical Sciences.

## Abbreviations

DLN: Delphian lymph node
PTC: Papillary thyroid cancer
SD: Standard deviation
